# Oncometabolites at the crossroads of genetic, epigenetic and ecological alterations in cancer

**DOI:** 10.1038/s41418-024-01402-6

**Published:** 2024-10-23

**Authors:** Letizia Lanzetti

**Affiliations:** 1 Department of Oncology, University of Turin Medical School, Turin, Italy; 2 Candiolo Cancer Institute, FPO-IRCCS, Str. Provinciale 142 km 3.95, 10060 Candiolo, Turin Italy

**Keywords:** Cancer metabolism, Cancer microenvironment

## Abstract

By the time a tumor reaches clinical detectability, it contains around 10^8^–10^9^ cells. However, during tumor formation, significant cell loss occurs due to cell death. In some estimates, it could take up to a thousand cell generations, over a ~ 20-year life-span of a tumor, to reach clinical detectability, which would correspond to a “theoretical” generation of ~10^30^ cells. These rough calculations indicate that cancers are under negative selection. The fact that they thrive implies that they “evolve”, and that their evolutionary trajectories are shaped by the pressure of the environment. Evolvability of a cancer is a function of its heterogeneity, which could be at the genetic, epigenetic, and ecological/microenvironmental levels [1]. These principles were summarized in a proposed classification in which Evo (evolutionary) and Eco (ecological) indexes are used to label cancers [1]. The Evo index addresses cancer cell-autonomous heterogeneity (genetic/epigenetic). The Eco index describes the ecological landscape (non-cell-autonomous) in terms of hazards to cancer survival and resources available. The reciprocal influence of Evo and Eco components is critical, as it can trigger self-sustaining loops that shape cancer evolvability [2]. Among the various hallmarks of cancer [3], metabolic alterations appear unique in that they intersect with both Evo and Eco components. This is partly because altered metabolism leads to the accumulation of oncometabolites. These oncometabolites have traditionally been viewed as mediators of non-cell-autonomous alterations in the cancer microenvironment. However, they are now increasingly recognized as inducers of genetic and epigenetic modifications. Thus, oncometabolites are uniquely positioned at the crossroads of genetic, epigenetic and ecological alterations in cancer. In this review, the mechanisms of action of oncometabolites will be summarized, together with their roles in the Evo and Eco phenotypic components of cancer evolvability. An evolutionary perspective of the impact of oncometabolites on the natural history of cancer will be presented.

## FACTS

- Oncometabolites induce modifications in the cancer microenvironment increasing cancer fitness by minimizing hazards and allowing exploitation of resources.
- Oncometabolites also induce variations in cancer cells (genetic and epigenetic), providing cancer cell-autonomous selectable advantages.
- Cell-autonomous and non-cell-autonomous oncometabolite-driven phenotypes cooperate to shape cancer evolvability as an “organoid”.

## Open questions

- Do oncometabolites recapitulate the effects of the upstream metabolic lesions?
- Can oncometabolites induce genetic variations in normal cells of the tumor microenvironment?
- Do oncometabolites play different roles in the natural history of cancer, e.g., in the initiation *vs*. the progression phase?
- Are the oncometabolite-induced phenotypes selected adaptively or exaptively?

## Oncometabolites and their origin

**Fig. 1:**
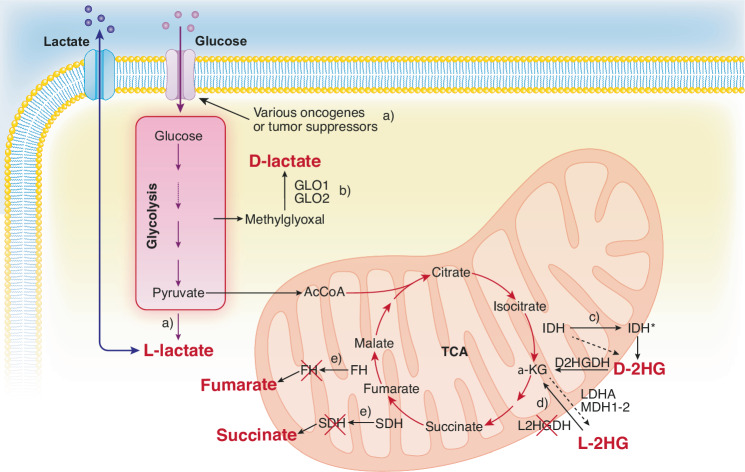
Origin of oncometabolites. **a**) Various oncogenes and tumor suppressors (e.g., RAS, MYC, PI3K, mTOR and p53) induce, via different mechanisms, increased glucose uptake and upregulated glycolysis, leading to increased L-lactate production. **b**) The methylglyoxal pathway is an offshoot of glycolysis, where methylglyoxal is transformed into D-lactate by the action of the enzymes glyoxalase 1 and 2 (GLO1 and GLO2). **c**) α-ketoglutarate (α-KG) is produced in the TCA by the catalytic action of isocitrate dehydrogenase 1/2 (IDH) on isocitrate. IDH can also generate, with low efficiency, D-2-hydroxyglutarate (D-2HG) (indicated by a dashed line). D-2HG can also be produced physiologically by the promiscuous activity of other enzymes (not shown, see main text). Mutations in IDH lead to the synthesis of gain-of-function IDH proteins (IDH*) capable of metabolizing isocitrate into D-2-hydroxyglutarate (D-2HG) with high efficiency. Note that the enzyme D-2-hydroxyglutarate dehydrogenase (D2HGDH) promptly convert D-2HG into α-KG, under physiological conditions. **d**) Physiologically, the promiscuous activity of lactate dehydrogenase A (LDHA) or of malate dehydrogenase 1/2 (MDH1-2) can convert α-KG into L-2HG (indicated by a dashed line). This effect is efficiently counteracted by L-2-hydroxyglutarate dehydrogenase (L2HGDH), whose decreased activity, in some cancers, leads to the accumulation of L-2HG. **e**) Loss-of-function mutations in one of the components of the succinate dehydrogenase (SDH) complex or in fumarate hydratase (FH) lead to the accumulation of succinate and fumarate, respectively. Abbreviations: TCA tricarboxylic acid cycle, AcCoA acetyl-coenzyme A, GLO1/GLO2 glyoxalase 1 and 2, α-KG α-ketoglutarate, IDH isocitrate dehydrogenase 1/2, IDH* mutated isocitrate dehydrogenase 1/2, D-2HG D-2-hydroxyglutarate, L-2HG L-2-hydroxyglutarate, LDHA lactate dehydrogenase A, MDH1-2 malate dehydrogenase 1/2, FH fumarate hydratase, SDH succinate dehydrogenase complex.

In cancer, metabolic pathways can be perturbed by genetic mutations, alterations in gene expression and/or protein modifications, leading to the accumulation of intermediate metabolites (Fig. 1), which are globally referred to as “oncometabolites”. Oncometabolites do not represent aberrant metabolites (with the partial exception of 2-hydroxyglutarate, to be described later). They are actually standard products of intermediate metabolism that physiologically participate to cellular homeostasis. In cancer, however, their accumulation at supraphysiological levels contributes to tumorigenesis by intersecting with the mechanisms of cancer evolvability through: (i) modification of cancer ecology (e.g., by induction of angiogenesis and suppression of the host immune response), (ii) alteration of the epigenetics of both cancer cells and “normal” adjacent cells, and (iii) induction of genetic alterations in cancer cells.

While many molecules have been proposed as oncometabolites, this review will focus on the paradigmatic examples of lactate (both L and D enantiomers), fumarate, succinate, and 2-hydroxyglutarate (2HG, both L and D enantiomers). These molecules result from alterations in energy-producing metabolism (glycolysis and the tricarboxylic acid cycle, TCA, Fig. 1) and substantial experimental evidence supports their role as bona fide oncometabolites.

### Alterations in glycolysis: lactate

L-lactate accumulates in several types of cancer as a result of the Warburg effect [4], where cancer cells preferentially reduce pyruvate to L-lactate (Fig. 1). While lactate production is typical under physiological conditions of hypoxia (for instance, during exercise), cancer cells produce lactate even in the presence of oxygen, hence the term “aerobic glycolysis”, a synonym of the Warburg effect. Traditionally, this “metabolic switch” to lactate production was attributed to cancer-specific mitochondrial disfunction. According to this view, the conversion of pyruvate to lactate, which yields 1 NAD^+^ per reaction, is crucial for supporting glycolysis (which consumes NAD^+^), and, therefore, ATP production when mitochondrial oxidative phosphorylation (OXPHOS) is limited. However, it is now understood that mitochondria in cancer cells are typically functional and actively involved in both efficient energy production and the generation of anabolic intermediates [4–6]. Indeed, mitochondrial OXPHOS appears to be indispensable for tumor survival, as evidenced by a large survey of mitochondrial DNA in tumors, which revealed that loss-of-function mutations in the cell respiration machinery, are under purifying selective pressure [7].

These observations raise important questions: what advantage does a cancer cell gain by activating aerobic glycolysis? What triggers the events leading to the Warburg effect? Some insights can be gained by closer inspection of the molecular players involved. Two of the most prominent molecular hallmarks of the Warburg effect are the increased production and presence of glucose and lactate transporters on the plasma membrane (PM) [5]. Consequently, cancer cells take up enormous amounts of glucose, fueling the glycolytic pathway. This might provide an advantage, as glycolytic intermediates feed numerous anabolic pathways, including: (i) the pentose-phosphate pathway, (ii) the hexosamine pathway, (iii) glycerol biosynthesis, and (iv) serine–glycine–one-carbon metabolism.

The question, however, remains: why not direct pyruvate, the end product of glycolysis, to the mitochondria for conversion into acetyl-coenzyme A (acetyl-CoA) to feed the TCA? One possibility is that in tumors with elevated glycolytic rates (due to enhanced glucose intake), the production of pyruvate might exceed the mitochondria’s capacity for oxidation, leaving no alternative but its conversion into lactate (to regenerate NAD^+^). Lactate is subsequently secreted and delivered to the liver through the Cori cycle, where it is converted back into glucose via gluconeogenesis. Alternatively, it can be utilized by neighboring cells or other cells throughout the body as a carbon source or to sustain energy production [8, 9]. However, pyruvate is as good a substrate for these purposes as lactate; hence the next question, why not simply secrete the excess pyruvate? Is the regeneration of NAD^+^ in the pyruvate-to-lactate conversion the advantage-conferring event? Alternatively, could lactate itself be an advantage-conferring metabolite? This possibility will be further discussed later on.

Compared to the alterations in the TCA cycle (see below), which are driven by specific genetic alterations, the metabolic reprogramming that culminates in the Warburg effects seems to be a common final outcome of many alterations. Several major oncogenic and tumor-suppressor pathways, including RAS, p53, MYC, PI3K, and mTOR, can induce this effect (Fig. 1). A detailed description of these mechanisms is beyond the scope of this review; thus, the reader is referred to excellent reviews on the subject [10, 11].

Interestingly, the D enantiomer of lactate also accumulates as the result of enhanced glycolytic metabolism. Physiologically, D-lactate accounts for only 1–5% of total lactate in the body, and is produced by carbohydrate-fermenting bacteria in the gastrointestinal tract or through the methylglyoxal (MG) pathway, an obligatory offshoot of glycolysis (Fig. 1) [12]. MG is primarily produced through non-enzymatic degradation of the glycolytic intermediate glyceraldehyde 3-phosphate. Being a highly reactive carbonyl species, MG can form adducts with proteins, DNA and GSH, and its accumulation is suspected to play a role in numerous pathological conditions, including neurodegenerative diseases, diabetes, and cancer [12]. In cancer, in particular, MG has been linked to initiation and progression, through cell-autonomous and non-cell-autonomous mechanisms [13, 14], therefore probably representing an oncometabolite on its own. For our present purposes, it is of relevance that MG is detoxified, in mammalian cells, through GLO1/2 glyoxalases, leading to the production of GSH and D-lactate (Fig. 1).

### Alterations in the TCA cycle: 2HG, fumarate and succinate

#### D-2HG and L-2HG

Isocitrate dehydrogenases (IDH1 and IDH2) catalyze the conversion of isocitrate into α-ketoglutarate (α-KG) in the TCA (Fig. 1). IDHs can also generate, with low efficiency, D-2-hydroxyglutarate (D-2HG) (Fig. 1). D-2HG can also be produced through the activity of 3-phosphoglycerate dehydrogenase (PHGDH) and hydroxyacid-oxoacid transhydrogenase (ADHFE1) (reviewed in [15]). Physiologically, D-2HG is rapidly converted to α-KG by the enzyme D-2-hydroxyglutarate dehydrogenase (D2HGDH). L-2HG is produced in the cell, starting from α-KG, through the spurious activity of malate dehydrogenases 1 and 2 (MDH1 and MDH2), and lactate dehydrogenase A (LDHA) in hypoxic and acidic conditions (Fig. 1) [16, 17]. Also in this case, the levels of L-2HG are kept low by the action of a specific dehydrogenase, L-2-hydroxyglutarate dehydrogenase (L2HGDH). The importance of the reactions executed by D2HGDH and L2HGDH is underscored by the existence of two genetic diseases, D-2-hydroxyglutaric aciduria 1 (D2HGA1, OMIM 600721) and L-2-hydroxyglutaric aciduria (L2HGA, OMIM 236792) due to biallelic loss-of-function of D2HGDH and L2HGDH, respectively, primarily characterized by neurological and developmental abnormalities. Thus, D2HGDH and L2HGDH detoxify D-2HG and L-2HG, respectively.

For our purposes, it is noteworthy that somatic mutations in IDH1 and IDH2 are found in several cancers – principally acute myelogenous leukemia (AML) and glioblastoma [18–20], but also cholangiocarcinomas and chondrosarcomas – and have proven to be effective therapeutic targets [21]. These mutations confer a neomorphic activity to IDH, disrupting the normal oxidative decarboxylation of isocitrate to α-ketoglutarate (α-KG) and promoting instead the reduction of α-KG to D-2HG (Fig. 1). In this case, it is likely that the activity of D2HGDH is overwhelmed by the activity of mutated IDHs, leading to the accumulation of D-2HG.

In human clear cell renal carcinoma, significant increases in the levels of L-2HG have been reported, associated to decreased levels of L2HGDH and loss-of-heterozygosity of its gene locus (Fig. 1) (reviewed in [22]). Thus, *L2HGDH* is a putative tumor suppressor gene and L-2HG a probable oncometabolite.

#### Fumarate and succinate

Germline mutations in the genes encoding succinate dehydrogenase (SDH) and fumarate hydratase (FH) are implicated in hereditary cancer syndromes [23–25] (Fig. 1). These mutations affect the *FH* gene or one of the four components of the SDH complex (also known as respiratory complex II) or the SDH complex assembly factor II, SDHFA2 (Table 1).

**Table 1:** Hereditary cancer syndromes leading to the accumulation of oncometabolites.

Gene	Syndrome	OMIM
*FH*	Hereditary leiomyomatosis and renal cell cancer (HLRCC)	150800
*SDHA*	Pheochromocytoma/paraganglioma syndrome 5 (PPGL5)	614165
*SDHB*	Pheochromocytoma/paraganglioma syndrome 4 (PPGL4)	115310
	Paraganglioma and gastric stromal sarcoma	606864
	Gastrointestinal stromal tumor (GIST)	606764
*SDHC*	Pheochromocytoma/paraganglioma syndrome 3 (PPGL3)	605373
	Paraganglioma and gastric stromal sarcoma	606864
	Gastrointestinal stromal tumor (GIST)	606764
*SDHD*	Pheochromocytoma/paraganglioma syndrome 1 (PPGL1)	168000
	Paraganglioma and gastric stromal sarcoma	606864
*SDHAF2*	Pheochromocytoma/paraganglioma syndrome 2 (PPGL2)	601650

Germline loss-of-function mutated alleles in the indicated genes are inherited and they predispose to the onset of cancer once somatic mutations occur in the other allele, according to the two-hit model. In the case of PPGL1, the heterozygous loss-of-function mutation is sufficient, due to maternal gene imprinting. The genes involved, the name of the syndrome and the OMIM (www.omim.org) number are included.

Inactivation of the SDH complex leads to the accumulation of succinate, while inactivation of FH results in the accumulation of fumarate. For detailed descriptions of the molecular genetics of these syndromes, the reader is referred to the OMIM database (https://www.omim.org/). However, it should be noted that it remains unclear why a “general” germline metabolic alteration leads to organ-specific tumorigenesis in these syndromes. The pre-existing cellular context, including both epigenetic and microenvironmental factors, likely plays a crucial, albeit not fully understood, role.

Perturbed levels of these oncometabolites might also be implicated in other types of cancer. For instance, D-2HG has been associated with sporadic breast and colon cancers [26–28]. In lung cancer patients, low expression levels of FH correlate with lymph node metastasis and overall poor survival [29]. Similarly, in prostate cancer, high levels of fumarate correlate with tumor aggressiveness [30]. Finally, succinate levels appear to be elevated in colorectal cancer tissues compared to surrounding normal tissues [31]. While the picture is by no means complete, these initial observations suggest a more pervasive role for the dysregulation of these oncometabolites in cancer. Advances in the metabolic profiling of cancer cells and tissues should help clarify this issue [32, 33].

## Oncometabolites: mechanisms of action

**Fig. 2:**
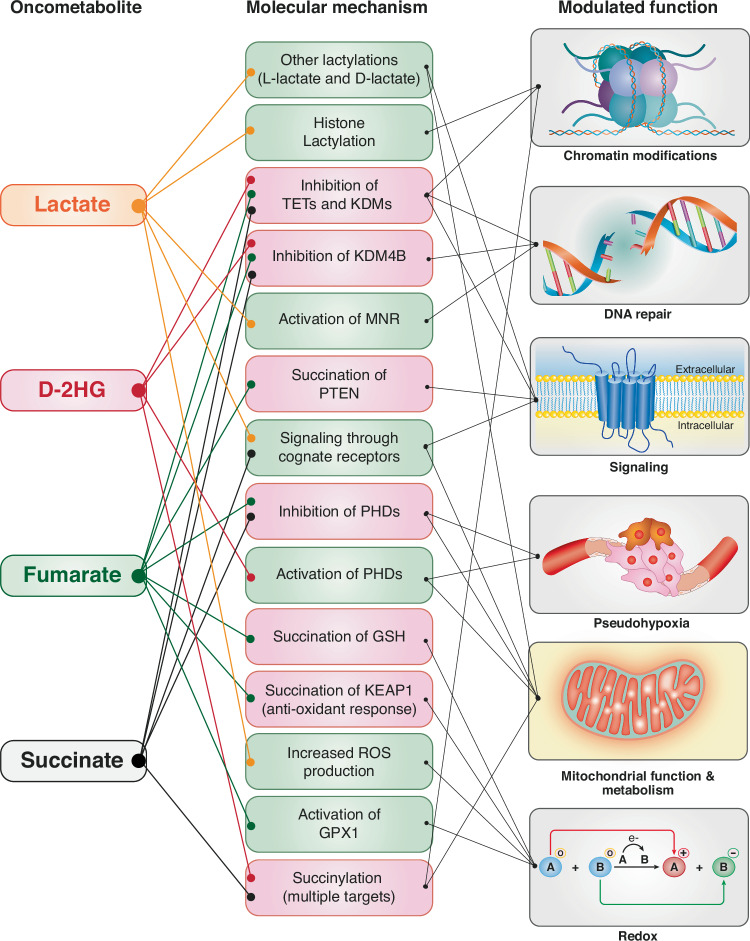
Molecular mechanisms of action of oncometabolites. The molecular mechanisms (middle column) through which oncometabolites (left column) modulate various cellular functions (right column) are depicted. In the “molecular mechanism” column, activating and inhibitory functions are boxed in green and red, respectively. The names of the oncometabolites are color-coded together with the connections to the molecular mechanism, to facilitate reading. Details are in the main text. Not all mechanisms/functions described for all oncometabolites are depicted.

Oncometabolites operate through various interconnected mechanisms that feed into one another, creating a highly complex network (Fig. 2). In addition, since oncometabolites are secreted, their molecular functions can influence both cancer cells and the tumor microenvironment.

### Epigenetic regulation of transcription

Oncometabolites can induce chromatin modifications, either directly or indirectly, leading to epigenetic regulation of gene expression patterns. For example, L-lactate can induce histone lactylation resulting in changes in transcription. L-lactate is first converted into lactyl-CoA by an unknown enzyme. The lactyl group is then transferred to the ε-amino group of lysine residues in histone proteins, in a reaction likely catalyzed by the histone acetyltransferase p300 [34–37]. The resulting phenotypes can be both cell-autonomous and non-cell-autonomous. In ocular melanoma cells, L-lactylation of histone H3 in the promoter region of the *YTHDF2* gene induces the expression of YTHDF2, an N^6^-methyladenosine (m^6^A) reader protein. YTHDF2 recognizes the m6A-modified *PER1* and *TP53* mRNAs, promoting their degradation, which results in increased proliferation and migration [38]. In the tumor microenvironment, histone L-lactylation in tumor-infiltrating myeloid cells (TIMs) promotes their immune-suppressive phenotype [39]. Specifically, histone lactylation in the promoter region of the methyltransferase-like3 gene (*METTL3*) upregulates the expression of this enzyme in TIMs [39]. METTL3, by inducing m^6^A modification of mRNAs, promotes the JAK1/STAT3 axis, enhancing the immunosuppressive function of TIMs [39].

Fumarate, succinate and D-2HG influence epigenetic reprogramming through a common mechanism involving the inhibition of a large class of epigenetic modifiers known as α-KG-dependent dioxygenases (α-KGDDs). These enzymes drive the oxidative hydroxylation of substrates through reactions that consume α-KG [40, 41]. Due to their structural similarity to α-KG, fumarate, succinate and D-2HG act as competitive inhibitors of α-KGDDs [40]. The two major types of α-KGDDs inhibited by these oncometabolites are TETs (Ten-Eleven Translocations) and KDMs (Jumonji domain-containing histone-lysine demethylases) [42–45], which catalyze the demethylation of DNA and histones, respectively. This inhibition leads to a hypermethylated phenotype, characterized by the altered expression of genes involved in DNA repair, apoptosis, cell cycle regulation, cell differentiation, and adhesion.

One outstanding issue concerns the identification of the “driver” epigenetic targets of the hypermethylated phenotype, as opposed to the multitude of “passenger” modifications induced by the inhibition of demethylases. In a recent effort, two such drivers were mechanistically characterized, for the hypermethylated phenotype induced by mutant IDH proteins. The methylation-induced disruption of a *PDGFRA* (platelet-derived growth factor alpha) insulator, in oligodendrocyte progenitor cells, allows an enhancer to aberrantly activate the transcription of the gene. In addition, methylation-induced silencing of the promoter of the tumor suppressor gene CDKN2A cooperated with the activation of PDGFRA to drive gliomagenesis. Thus, DNA hypermethylation, driven by IDH mutant proteins, activates an oncogene and represses a tumor suppressor gene [46].

Finally, the significance of TET and KDM inhibition by oncometabolites in tumorigenesis is underscored by the observation that these families of epigenetic modifiers are themselves frequently mutated in various types of cancer (reviewed in [40]).

### Other oncometabolite-dependent modifications

The impact of L-lactylation extends beyond histone modification, with nearly a thousand L-lactylated proteins identified [47]. Among these targets, lactylation of adenylate kinase 2 has been shown to reduce its activity, resulting in energy imbalances that promote the proliferation of hepatocellular carcinoma cells [47].

Interestingly, another lactate-based modification, D-lactylation, exists. Protein D-lactylation derives from the non-enzymatic acyl transfer of the D-lactate moiety to lysine residues [35]. In details, methylglyoxal (Fig. 1) is conjugated to GSH by the enzyme GLO1, yielding lactoylglutathione (LGSH). LGSH is then hydrolyzed by GLO2, producing GHS and D-lactate. However, the D-lactate moiety of LGSH can also be transferred, through non-enzymatic acyl transfer, to protein lysine residues, yielding D-lactylation. Approximately 350 proteins carrying this modification have been identified. Among them, glycolytic enzymes are primary targets, resulting in a global decrease of glycolytic output [35].

Fumarate and succinate (through succinyl-CoA) can also trigger the modification of other biological molecules in the reactions known as succination and succinylation, respectively (reviewed in [48, 49]).

Fumarate spontaneously reacts with the thiol group of cysteines, forming fumarate-cysteine adducts, in a chemical reaction termed succination. One protein that undergoes succination is KEAP1, the substrate recognition subunit of an E3 ubiquitin ligase complex that regulates NRF2, an important transcription factor in the antioxidant response. Succination inactivates KEAP1, leading to NRF2 stabilization and activation of the antioxidant response [50]. Other proteins inhibited by succination include the tumor suppressor PTEN [51] and gasdermin D, with inhibition of the latter leading to the suppression of pyroptotic cell death [52]. Additionally, fumarate can induce succination of GSH, leading to its depletion and disruption of the redox balance [53, 54]. A comprehensive review of the role of succination in physiology and pathology can be found in [48].

Succinylation is a post-translational modification whereby a succinyl moiety is transferred from succinyl-CoA to the ε-amino group of lysine residues, through enzymatic and non-enzymatic reactions (reviewed in [49]). Succinyl-CoA is produced in the cell starting from succinate and other metabolites, such as α-KG. It is, therefore, predicted that loss-of-function of SDH genes, which leads to the accumulation of succinate, will cause an increase in succinylation. Succinylation is involved in chromatin regulation and the modulation of protein activity, particularly in the mitochondria, with implications for cancer and other diseases (see for instance [55–57]). However, in cancer, the relevance of succinylation has been linked to alterations in succinylating (KAT2A, CPT1A, OXCT1) or de-succinylating enzymes (SIRT5, SIRT7). Therefore, it remains unclear whether an increase in succinate levels alone is sufficient to determine a cancer-relevant increase in succinylation. This possibility is supported by the finding that D-2HG (produced by mutated IDH1-2) competitively inhibits SDH, resulting in the accumulation of succinyl-CoA and hyper-succinylation of mitochondrial proteins, ultimately leading to apoptosis resistance, a hallmark of cancer [58]. This finding underscores how succinylation might represent a common mechanism through which some oncometabolites exert their effects.

### Receptor-mediated signaling

Cells display receptors on their surface, belonging to the G protein-coupled receptor (GPCR) superfamily, which can bind oncometabolites and transduce intracellular signals. Hydroxycarboxylic acid receptor 1 (HCAR1) and succinate receptor 1 (SUCNR1) are high-affinity receptors for lactate and succinate, respectively [59, 60]. In addition, oxygen-dependent G protein-coupled receptor 1 (OXGR1) binds α-KG, although whether it also binds D-2HG remains unclear [60].

The interaction between oncometabolites produced by cancer cells and their cognate receptors can trigger a series of paracrine and autocrine events. For instance, succinate secreted by lung cancer cells can engage SUCNR1 on macrophages, stimulating their polarization into tumor-associated macrophages (TAMs). These TAMs, in turn, secrete cytokines, in particular IL6, which promotes cancer cell migration, epithelial-to-mesenchymal transition (EMT), and metastasis [61].

In CD8 + T lymphocytes, the autocrine engagement of SUCNR1 by succinate is required to sustain anti-tumor cytotoxic activity [62]. Here, a remarkable interplay between oncometabolites determines a cancer advantage. Lactate produced by cancer cells rewires metabolism in CD8+ cells by decreasing pyruvate carboxylase (PC) activity, which is crucial for the anaplerotic replenishment of the TCA cycle and the production/secretion of succinate. This downregulation of PC interferes with the cytotoxic-promoting action of the autocrine succinate/SUCNR1 axis in CD8+ cells [62].

Finally, interactions between oncometabolites and their receptors can have significant effects on the host-cancer relationship. For instance, tumor-produced lactate activates, via HCAR1, major catabolic effects in adipose and muscle cells, leading to cancer cachexia [63].

The major implication of the discovery of the signaling ability of oncometabolites is that the presence of cognate receptors argues that they must be part of physiological mechanisms that were selected for in evolution. Thus, oncometabolites might physiologically function as cytokines and be involved in a number of cellular and organismal responses [64]. Indeed, there is evidence supporting their involvement in the regulation of the immune response, energy metabolism, neurogenesis, and organ and tissue homeostasis [59, 65–71]. These findings suggest that the cancer-related functions of oncometabolites might represent, at least in part, an exaggeration (or ectopic activation) of their physiological functions. This is perhaps not surprising, as even the “truest” metabolic hallmark of cancer, aerobic glycolysis, can occur under physiological conditions in connection with the execution of diverse functions in neurons, endothelial cells, monocytes, neural crest cells, pluripotent stem cells, and presomitic mesoderm [72–76].

### Pseudohypoxia

In addition to regulating α-KGDDs involved in epigenetic reprogramming, the oncometabolites D-2HG, fumarate and succinate also modulate the activity of another class of α-KGDDs, the HIF-prolyl 4-hydroxylases (PHDs) [40]. These enzymes modify the hypoxia-inducible factors HIF1α and HIF2α (HIFα) [77] by hydroxylation of proline residues, which facilitates their ubiquitination and proteasomal degradation [77]. The inhibition of PHDs by fumarate or succinate leads to the stabilization of HIFα, which impacts angiogenesis and glucose metabolism [78, 79]. This phenomenon is termed pseudohypoxia as it mimics the HIFα-dependent hypoxia response in a non-hypoxic environment. In addition, it represents a convergence point of some oncometabolite-related responses, since it phenocopies key aspects of the Warburg effect, which are also largely mediated by activation of HIFα signaling under normoxic conditions.

Interestingly, the situation with D-2HG is somewhat different. Although D-2HG inhibits α-KGDDs of the TET and KDM families (as mentioned above), it stimulates the activity of PHDs, thereby blunting HIFα-mediated responses [80]. This effect might synergize with another D-2HG-mediated response. Specifically, D-2HG produced by cancer cells can be taken up by CD8 + T cells, leading to inhibition of their proliferation, cytotoxicity and interferon-γ signaling. This inhibition is mediated by direct suppression of lactate dehydrogenase (LDH), the enzyme responsible for the conversion of pyruvate into lactate, leading to increased mitochondrial respiration and decreased glycolysis. This switch in metabolism directly contributes to the observed effects of D-2HG on CD8 + T cells [81]. Effector CD8 + T cells, once activated by antigen recognition, depend on high glycolytic flux to meet their bioenergetic and anabolic needs [82]. Thus, the secretion of the oncometabolite D-2HG by cancer cells might serve as a mechanism to evade the immune response by altering the metabolic environment of immune cells.

An interesting twist is provided by a study showing that subcutaneous injection of lactate into tumor-bearing animals reduces tumor growth [83]. This effect was linked to lactate-induced increased stemness of tumor-infiltrating CD8 + T cells. While these results are at odd with the bulk of the evidence supporting an immuno-suppressive role for lactate in tumors (see later), they provide an interesting system to study the possible antagonism between D-2HG and lactate on the survival and the function of tumor-infiltrating CD8 + T cells, in which threshold-controlled effects of the two oncometabolites can tip the balance towards an immune-suppressive *vs*. an immune-promoting phenotype. Indeed, the issue of the interplay of various oncometabolites in cancer remains a largely unresolved one. This is important since the metabolic alterations leading to the accumulation of oncometabolites should not be viewed as “stand alone” occurrences, but rather as triggers of major metabolic reprogramming with projected impact on the levels of several oncometabolites (see, for instance [48], for the reprogramming induced by defects in FH).

### Effects on homology-dependent DNA repair

The inhibition of α-KGDDs by fumarate, succinate and D-2HG also influences DNA repair mechanisms, specifically homology-dependent repair, through an epigenetic mechanism. These oncometabolites inhibit the histone demethylase KDM4B, leading to aberrant H2K9 hypermethylation, which impedes the proper recruitment of factors, such as TIP60 and ATM, required for the execution of homology-dependent repair [84, 85]. This impairment of DNA repair can lead to genomic instability, suggesting a direct role of these oncometabolites in determining cancer genetics.

These findings have implications not only for cancer cell-autonomous alterations, but also potentially for non-cell-autonomous mutational landscapes. Since oncometabolites are actively secreted, they could induce genomic instability in stromal cells. There is some evidence suggesting that tumor stromal cells harbor cancer-relevant mutations, both in experimental models and actual human cancers. However, this evidence has been disputed and attributed to technical and/or methodological artifacts [86]. A recent paper employing single-cell multi-omics sequencing has provided more convincing evidence that stromal cells (fibroblasts, endothelial, and immune cells) surrounding colon cancers harbor prevalent genetic changes [87]. How cancer cells might induce mutations in the surrounding stroma remains undetermined, and various scenarios can be envisaged. Among these, the ability of certain oncometabolites to induce genetic changes represents an intriguing possibility.

In contrast to fumarate, succinate and D-2HG, lactate exerts a positive effect on homology-dependent DNA repair. The MRN complex – composed of MRE11, RAD50, and NBS1 – binds to DNA and executes the end resection of damaged DNA. Lactylation controls the MRN complex through a two-fold mechanism. Lactylation of NSB1 is required for proper complex assembly, while lactylation of MRE11 augments complex binding to DNA and its activity [88, 89]. Paradoxically, an enhanced homology-dependent repair capability might confer a cancer advantage upon exposure to therapy, since it increases resistance to cancer drugs that induce DNA damage [88, 89].

### Redox alterations

Alterations in redox homeostasis, leading to increased production of reactive oxygen species (ROS), are common in cancer and have been reviewed extensively elsewhere [10, 90]. Oncometabolites can directly impact ROS production and redox homeostasis through various mechanisms.

There are a few reports suggesting that lactate accumulation leads to ROS production by unknown mechanisms [91, 92]. Interestingly, lactate can also increase ROS production by direct non-enzymatic mechanisms, through the Fenton reaction [93]. In one example of how a lactate-ROS feed-forward circuitry, between cancer and stromal cells, can contribute to cancer progression, it was shown that lactate, secreted by cancer cells, is taken up by cancer-associated fibroblasts (CAFs) where it causes, with unknown mechanism, a ROS upsurge, leading to NFkB activation. This, in turn, stimulates the transcriptional activation and the secretion of HGF, which stimulates the receptor tyrosine kinase MET on cancer cells, leading to resistance to tyrosine kinase inhibitors [94].

More substantial evidence exists for fumarate. The impact of fumarate-dependent succination on the KEAP1-NRF2 axis, leading to antioxidant responses [50, 95], and on GSH, leading to depletion of this pivotal ROS scavenger [53, 54] has already been discussed. In addition, fumarate can bind to and activate the ROS-scavenging enzyme GPX1 (glutathione peroxidase 1), promoting tumor cell growth and survival [96]. It is perhaps surprising that fumarate can control antioxidant responses both in a positive and negative manner. However, this apparent contradiction can be resolved when one considers that cancer cells mount antioxidant responses to protect themselves from the harmful effects of increased ROS production [97, 98]. In addition, a moderate level of ROS is needed to maintain cellular homeostasis [99, 100]. Thus, if a cancer cell can protect itself from excess ROS, by mounting an effective antioxidant response, it might receive additional benefits from increased ROS levels.

This concept is exemplified by the so-called “reverse Warburg effect” [101, 102], which involves a feedback loop between ROS and lactate production in cancer and stromal cells. Cancer cells can induce stromal cells to undergo metabolic reprogramming, resulting in a glycolytic phenotype, through converging mechanisms. ROS released from tumor cells induce oxidative stress in CAFs, leading to HIF-1α activation and enhanced glycolysis. This effect is reinforced by the fact that glucose consumption by the cancer epithelial component leads to glucose depletion in the tumor microenvironment and a decrease of ATP in the stromal cells, further stimulating the HIF-1α-mediated response. The net result is that stromal cells (particularly CAFs) secrete high amounts of lactate that is taken up by cancer cells [103, 104]. Cancer cells then exploit lactate as a high-energy carbon source that sustains their energy and lipid production needs. Indeed, in non-small cell lung cancer (NSCLC), lactate is a prominent respiratory fuel that exceeds even the contribution of glucose [105].

This latter observation warrants further discussion, as this property of lactate might not be unique to cancer. In mice, lactate was demonstrated to be a primary carbon source for the TCA cycle, as shown in experiments utilizing ^13^C-lactate, where TCA cycle intermediates were extensively labeled with ^13^C in all tissues [106]. This finding suggests that the conversion of pyruvate into lactate might be a more widespread metabolic characteristic in physiology than previously thought. In this scenario, glucose could systemically feed the TCA cycle through secreted lactate, potentially allowing glycolysis and the TCA cycle to be uncoupled. Cells that rely on circulating lactate to fuel the TCA cycle might meet their energy demands without needing to carry out glycolysis, which could then be locally activated to support anabolic processes when cells need to proliferate or restore local homeostasis. The situation might be even more complex, as a recent study demonstrated that lactate can directly stimulate the electron transport chain and energy production, independently of its metabolism – a property shared by both L- and D-lactate [107].

## Oncometabolites: Evo and Eco phenotypic intersections

Significant effort is being devoted to understanding the interactions between the phenotypic and molecular aspects of cancer-intrinsic and cancer-extrinsic events that shape the evolutionary trajectory of cancers. While it is reasonable to assume that cancer cell-autonomous events are paramount in the initial phases of cancer initiation, the disease eventually evolves into an ecosystem where the environment provides resources to the cancer but also presents hazards.

Cancer cells can hijack normal cells to receive resources (including metabolic ones), promote neo-angiogenesis, and activate motility programs including metastasis. These interactions are not necessarily parasitic in nature, since cancer cells can provide fitness-enhancing stimuli to normal cells, establishing a mutualistic relationship that underlies the co-evolution of the cancer and the normal compartment in the ecosystem. This reciprocal relationship results in the emergence of a “cancer organoid” – a unit of selection, evolutionarily speaking. However, hazards can arise within this ecosystem, resulting from the competition for resources (nutrients, oxygen) between the stromal components and the cancer cells, as well as from clear-cut examples of predation, particularly from the immune system.

Throughout the natural history of an individual cancer, the quality and quantity of resources available to a cancer, as well as the hazards that threaten its survival are constantly changing. The strategies employed by cancer cells to cope with hazards (or to exploit resources) can, therefore, vary during the different stages of tumor development – from the initiation phase and the growth of a clinically evident tumor to the metastatic stage and even the development of resistance to therapy [11].

For the purposes of this review, the central question is: What role do oncometabolites play in this complex intersection of the various phenotypes that are selected as advantage-conferring during the natural history of cancer?

### The Evo component

The ability of oncometabolites to induce epigenetic modifications and genomic instability (in the case of fumarate, succinate and D-2HG) indicates that they can be sources of Evo variability in cancer ecosystems. Although this field is still in its infancy and evidence is limited, some aspects are worth discussing:

The oncometabolite-induced Evo variability is potentially selectable in a cancer-cell-autonomous fashion. In the next paragraph the evidence suggesting that some oncometabolites might be involved in conferring a proliferative advantage during cancer initiation will be discussed. Conversely, at later stages of cancer development, the role of oncometabolites is evident and largely attributable to their effects on the tumor microenvironment (see below). It is worth mentioning that the “mutational” (genetic/epigenetic) impact of oncometabolites does not need to be limited to “driver” mutations to be selectable. “Passenger” mutations (genetic/epigenetic), which are generally considered less relevant for cancer progression or subject to weak purifying selection [108], might confer proliferative advantages when combined [109, 110]. These passenger mutations, much like polymorphic variability in a population, may also serve as a form of insurance against catastrophic changes in environmental conditions, such as those induced by therapy. For instance, lactylation-driven epigenetic modifications confer resistance to the chemotherapy drug temozolomide in glioblastoma [111].

Evo variability is not necessarily confined to cancer cells. The extent of oncometabolite-induced genetic/epigenetic alterations in stromal cells remains to be fully established. One outstanding question is whether the concentration of oncometabolites in the interstitial fluid (and their uptake by stromal cells) is sufficient to induce epigenetic or genetic changes in the receiving cell. However, it is clear that the phenomenon can occur, at least in certain circumstances, as exemplified by the above-mentioned effects of histone lactylation on the immunosuppressive phenotype of TIMs [39].

### The Eco component

**Fig. 3:**
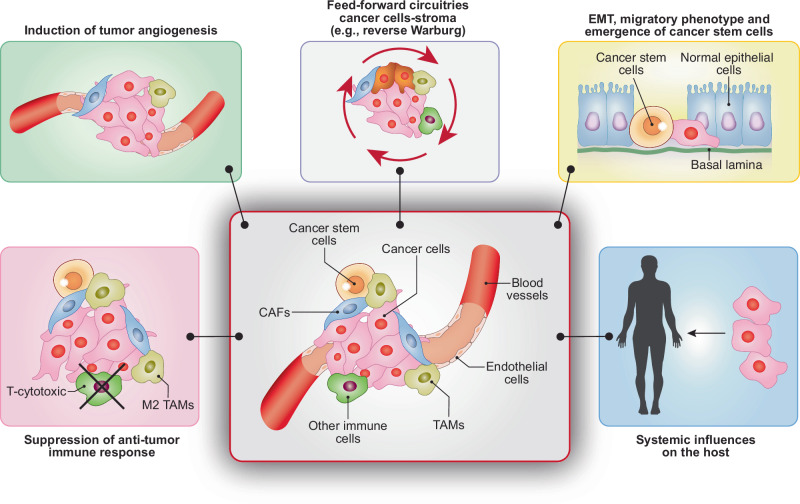
Oncometabolites and Eco (environmental) phenotypes. Some of the tumor-relevant phenotypes elicited by oncometabolites through their action on the tumor microenvironment or on the host environment are depicted. Details are in the main text. Abbreviations: M2 TAMs tumor-associated macrophages M2 type, TAMs tumor-associated macrophages, CAFs cancer-associated fibroblasts.

The impact of oncometabolites on tumor-associated phenotypes has been extensively analyzed within the context of the tumor microenvironment. The existing literature is vast and, at times, contradictory. This inconsistency is not surprising, given that oncometabolites are also produced in physiological settings and that much of the evidence was obtained through in vitro studies. In such studies, it can be difficult to differentiate between physiological and pathological effects, which might depend on oncometabolite concentration and the specific cellular context. Nevertheless, there are numerous tumor microenvironment-dependent phenotypes controlled by oncometabolites, through the above-mentioned molecular mechanisms. These phenotypes include tumor angiogenesis, immune evasion, and acquisition of migratory abilities, frequently connected to EMT. Although not exhaustive, the following figure summarizes the general principles (Fig. 3), with references to detailed reviews for more in-depth accounts.

- *Lactate*. Lactate plays a multifaceted role in the tumor microenvironment. It stimulates neo-angiogenesis within tumors [112–114] and also affects the tumor immune infiltrate through several mechanisms, contributing to immune evasion: (i) It decreases the activity of cytotoxic T cells and natural killer cells, while exerting limited or no effect on immunosuppressive cells (e.g., T_reg_ cells), (ii) It induces the polarization of tumor-associated macrophages (TAMs) towards the M2 phenotype which is associated with anti-inflammatory actions, (iii) It interferes with the function of antigen-presenting cells [115–117]. Lactate also promotes cancer cell invasion by stimulating stromal cells to produce pro-angiogenic factors, pro-migratory cytokines, and adhesion molecules [117, 118]. Consistently, reduced lactate production by LDHA knockdown, prevents metastatic spreading in a breast cancer mouse model [119], in line with the correlation between high lactate concentration in tumors and decreased patient survival [120]. For a comprehensive review of the biological effects of lactate in physiology and diseases, please see [121].

- *D-2HG, fumarate and succinate*. Succinate and fumarate can induce tumor angiogenesis via pseudohypoxia [22, 78].

In addition, the three oncometabolites also affect the anti-tumor immune response. D-2HG dampens cytotoxic T cell activity [81, 122] and contributes to creating a tumor macrophage-dependent immunosuppressive environment [123]. Interestingly, in the case of D-2HG, Evo (cell-autonomous) components are also pivotal in determining the immunosuppressive environment. A recent study highlighted how mutant IDH proteins dampen the activation of a program of immune-stimulatory type I interferon (IFNβ) and viral response genes in the tumor cells, through the hypermethylation and silencing of the cytosolic dsDNA sensor (cGAS) locus, therefore impairing innate immune sensing [124]. Succinate promotes macrophage polarization towards the immunosuppressive M2 phenotype [61]. Fumarate suppresses the anti-tumor activity of cytotoxic T cells [125]. Interestingly, fumarate can also trigger the release of mitochondrial DNA in the cytosol activating the cGAS-STING-TBK1 pathway, leading to the activation of the innate immune response [126]. How this latter effect might impact on tumorigenesis or on the modulation of the tumor immune microenvironment remains to be elucidated.

Finally, D-2HG and fumarate induce EMT [27, 127], a finding that will be discussed in the next section. For further details on the phenotypes induced by TCA-derived oncometabolites, refer to [15, 32, 128–130].

### Phenotypic intersections

**Fig. 4:**
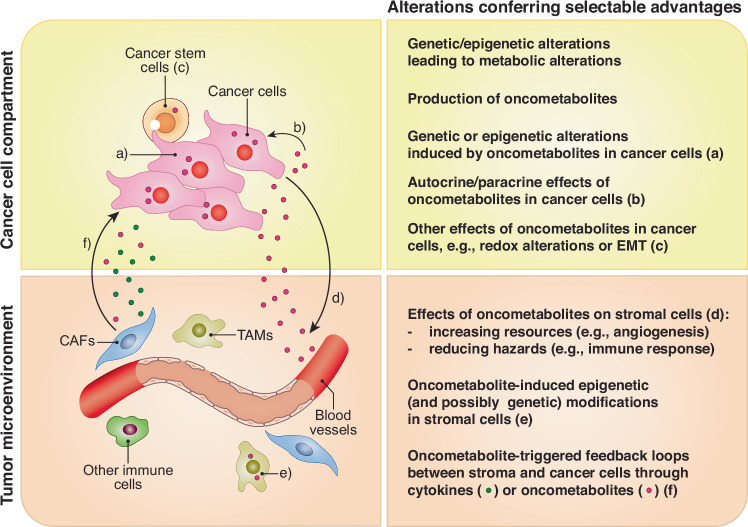
Integration of effects of oncometabolites (and their upstream genetic alterations) in cancer evolvability. In the left panels, a cancer is depicted with its two major components (cancer cells and tumor microenvironment) arbitrarily separated. The communication between these two compartments occurs via various mechanisms. Mechanisms mediated by oncometabolites are shown in the illustration. In the right panels, some selectable advantages conferred by oncometabolites are shown in the two compartments. Not all the actions of oncometabolites are depicted. Note that the various phenotypes might be selected for during different phases of tumor development. Details are in the main text. Abbreviations: CAFs cancer-associated fibroblasts, TAMs tumor-associated macrophages, EMT epithelial-mesenchymal transition.

The magnitude of Eco-relevant phenotypes elicited by oncometabolites, the complex inter-relationships and feedback mechanisms among these phenotypes, and the emerging role of oncometabolites as agents of Evo variability, raises the question of how all this information can be integrated for predicting tumor behavior and, possibly, guiding therapeutic interventions. For successful integration, it is essential to include data on the metabolic status of the tumor (Fig. 4). However, an intrinsic limitation of current technology is the scarcity of quantitative methods to measure in vivo metabolic states, changes, and oncometabolite concentrations, over space and time.

Efforts to overcome these limitations are being implemented, as for instance the use of Raman micro-spectroscopy for the detection of oncometabolites in living cells in vivo and animal tissues ex vivo [131]. One interesting concept emerging from these efforts is that of “metabolic zonation”, i.e., the presence of regions, within a tissue, in which local conditions (e.g., the proximity to blood vessels) shape the metabolic landscape. The concept was firstly established in liver physiology and pathology (for a review see [132]) and subsequently extended to cancer [133]. Metabolic zonation contributes to metabolic cancer heterogeneity and might further contribute to metabolism-dependent cancer evolvability.

Another promising approach to overcoming this challenge is the development of predictive models. For example, a network comprising 13 genes, 17 enzymes, and 23 metabolites was created and used to quantify the driving forces behind cancer metabolism dynamics [134]. This analysis led to the definition of “metabolic cancer landscapes” and the identification of key regulatory interactions that drive shifts between them. Although this approach is in its early stages, it holds potential for predicting the effects of environmental changes (such as the concentrations of key metabolites/resources) and of therapeutic metabolic interventions (hazards).

## A cancer evolutionary perspective: adaptation or exaptation?

The reviewed evidence overwhelmingly demonstrates that oncometabolites intersect with many cell-intrinsic and cell-extrinsic cancer-relevant phenotypes, justifying continued efforts to exploit oncometabolites and, more broadly, metabolic vulnerabilities in cancer treatment (reviewed in [32, 135]).

From the perspective of cancer biology, while there is little doubt that oncometabolites play a major role in the late phases of the natural history of cancer, corresponding to what is generally called cancer “progression”, one unresolved question is whether they induce advantage-conferring events during tumor initiation. This question has been debated mostly in relation to the Warburg effect and to the hypothesis that lactate production might represent the true “purpose” of aerobic glycolysis.

The issue is complex, as oncometabolite production is inextricably linked to upstream metabolic alterations and is further complicated by the fact that when a tumor becomes clinically evident, and thus amenable to study, at least two-thirds of its natural history have already elapsed. To gain further insights, we must focus on the initial phases of carcinogenesis, when the unit of selection is the individual cancer cell and any advantage-conferring event must be cell-autonomous. In this phase, we can entertain two scenarios:

- *Adaptive evolution:* The oncometabolite confers a direct advantage, behaving as a “carcinogen”. In this case, the selection of the advantage-conferring event could be considered adaptive.

- *Exaptive evolution:* The metabolic alteration confers the cell-autonomous advantage. How this advantage is achieved is, in large part, speculative. Normal cells are generally expected to possess primarily catabolic/energetic metabolism to support their various functions. In contrast, cancer cells typically have high metabolic demands on both the anabolic (biomass production) and catabolic (production of energy) fronts. The metabolic alterations discussed here share the common property of increasing the availability of metabolic intermediates that can be used for anabolic processes, while energy demands can be met through various circuitries, such as aerobic glycolysis, anaplerotic reactions in the case of TCA cycle alterations, or decreasing the energy requirements by switching off ATP-costly activities [136]. In this context, the production of oncometabolites might represent an obligatory consequence of these metabolic alterations rather than an advantage-conferring trait. In other words, they might represent what Gould and Lewontin define as “spandrels” [137]. However, oncometabolites might confer an advantage at later phases of cancer development, when the unit of selection shifts from the individual cancer cell to the “cancer organoid”, which has increasing need to obtain resources and avoid hazards. This situation exemplifies what Gould and Vrba define as exaptation [138].

An acid test to differentiate among these possibilities is to answer the question: Are oncometabolites alone capable of transforming cells in culture or inducing tumors in experimental animals?

In the case of D-2HG, the answer is probably yes. Exposure of leukemic cells to D-2HG promoted reversible cytokine independence and a differentiation block: two characteristics of leukemogenesis [139]. In another study, it was shown that D-2HG could induce monocytic leukemia in vivo, in animals [140]. The effect was observed only in mice harboring an additional genetic lesion, namely overexpression of HOXA9 [141], and not in wild-type animals. Thus, D-2HG alone did not induce transformation. This finding is not surprising given the consolidated evidence indicating that cellular transformation requires multiple hits, and that IDH mutations alone are not sufficient to induce leukemia [142, 143]. Furthermore, the study found that the effects of the mutant IDH1 protein were stronger than those achieved by D-2HG alone, at comparable levels of D-2HG [140]. This suggests that D-2HG-independent oncogenic functions of mutant IDH1 are likely required in addition to D-2HG. In summary, while D-2HG alone may not be sufficient for cancer initiation, its production might be necessary as part of adaptive selection.

In the case of fumarate and succinate, there are no reports describing cell transformation or tumorigenesis in animals induced by these oncometabolites. However, one could argue that the direct effect of fumarate on the activity of the potent tumor suppressor PTEN [51] suggests a possible role as a cancer initiator.

It is also worth noting that both D-2HG and fumarate induce EMT [27, 127]. EMT is a complex process in which sessile epithelial cells switch to a migratory mesenchymal-like state [144]. In cancer, EMT is associated with the acquisition of invasive and metastatic capabilities, cancer stem cell-like properties, and therapy resistance [145, 146]. Of these phenotypes, the ability of EMT to promote the emergence of cancer stem-like cells appears to be the most relevant to tumor initiation.

In the case of lactate, two recent papers have shown that lactylation hinders the transcriptional activity of p53 and that lactate can remodel the anaphase-promoting complex (APC/C) through binding/inhibition of SENP1, a SUMO protease that controls the activity of APC/C [147, 148]. Both findings suggest a potential direct role of lactate in tumor initiation. However, there is no evidence in the literature of lactate-induced cell transformation in vitro or tumorigenesis in vivo. This stands in contrast to the extensive literature on the cancer-related effects of lactate: the key “lactate AND cancer” yielded more than 28,000 hits in PubMed. Therefore, it is likely that lactate production is not a selected advantage in the initial cell-autonomous phase of cancer growth, implying that metabolic alterations upstream of its production are more relevant during this phase. Nevertheless, the production of lactate appears to be crucial at later stages of cancer development, when non-cell-autonomous, microenvironmental/ecological events might confer an advantage to the cancer organoid. According to this perspective, the selective advantage provided by lactate would be exaptive: a process that requires millions of years in evolution but could occur in years in the compressed evolutionary timeframe of a cancer.

## Concluding remarks

An Evo-Eco outlook appears indispensable to understand the impact of oncometabolites on cancer evolvability. Historically, oncometabolites have been studied mostly in connection with their ability to induce advantage-conferring alterations in the cancer microenvironment. More recently, their ability to act as agent of epigenetic and genetic alterations has come into focus. This delineates an extremely complex scenario in which cell-autonomous and non-cell-autonomous effects intersect, in a complex environment in which metabolic reprogramming (induced by the oncometabolites or by the upstream metabolic alterations) increasingly derails cellular homeostasis. Many questions, some of which are reported at the beginning of this review, remain: the answers will provide the necessary theoretical background for the development of interventional strategies.
